# Eliminate the In Vivo Digestibility Requirement for Protein Content Claims in North America to Align Consumer Purchasing Behavior with Dietary Guidelines

**DOI:** 10.1016/j.cdnut.2025.107627

**Published:** 2025-12-25

**Authors:** Joseph Manuppello, Christopher D Gardner, Anna Herby, Elaine S Krul, Christopher PF Marinangeli, Amanda Gomes Almeida Sá, Mingyang Song

**Affiliations:** 1 Physicians Committee for Responsible Medicine, Washington, DC, United States; 2 Stanford Prevention Research Center, Department of Medicine, School of Medicine, Stanford University, Palo Alto, CA, United States; 3 EKSci, Limited Liability Company (LLC), St. Louis, MO, United States; 4 Protein Industries Canada, Centre for Regulatory Research and Innovation, Regina, Canada; 5 Department of Biochemistry, Memorial University of Newfoundland, St. John’s, NL, Canada; 6 Department of Food and Human Nutritional Sciences, Richardson Centre for Food Technology and Research, University of Manitoba, Winnipeg, MB, Canada; 7 Departments of Epidemiology and Nutrition, Harvard T.H. Chan School of Public Health, Boston, MA, United States; 8 Clinical and Translational Epidemiology Unit, Massachusetts General Hospital and Harvard Medical School, Boston, MA, United States

**Keywords:** 3Rs, amino acids, animal testing alternatives, dietary protein, digestibility, food regulations, in vitro techniques, plant-based, protein quality, PDCAAS

## Abstract

A roundtable discussion, held on 10 December, 2024, addressed requirements for protein quality assessment in United States and Canadian food labeling regulations, focusing on concerns with the protein digestibility-corrected amino acid score (PDCAAS), which includes an in vivo rat assay to determine true fecal protein digestibility. Because animal proteins tend to score higher, the PDCAAS disadvantages nonanimal foods in substantiating protein content claims despite dietary guidelines recommending increased intake of plant proteins. In addition, the use of animal testing raises ethical concerns for some consumers. Roundtable participants weighed the benefits and costs of requiring the PDCAAS and discussed alternative regulatory approaches to better promote human health, prevent chronic disease, replace animal testing, and support sustainable food production. Options included relying solely on the amount of protein per serving, correcting only for the amino acid score, using fixed coefficients of digestibility or in vitro assays to determine digestibility, and incorporating measures that reflect human health outcomes and environmental impact. Several in vitro methods, such as the pH-drop and pH-stat methods, were identified as promising candidates for regulatory acceptance. The consensus was that for foods that do not address special needs, relying solely on the amount of protein to substantiate content claims is appropriate for populations who already consume protein in excess of reference values from varied sources. This approach, already used in other high-income jurisdictions, allows more plant-based foods to qualify for protein claims while avoiding animal testing. Moving away from the in vivo derived PDCAAS would reduce existing regulatory barriers, better align with current dietary guidelines, and promote increased intake of plant-based foods, thereby improving public health and sustainability.

## Introduction

Protein quality refers to the ability of dietary proteins to meet the human body’s needs for metabolism, maintenance, and growth by supplying all indispensable amino acids (IAAs) [1]. United States and Canadian food labeling regulations currently require that protein quality be assessed whenever a content claim is made [2,3]. As a measure of protein quality, both jurisdictions accept the protein digestibility-corrected amino acid score (PDCAAS), which is based on the lowest ratio of an IAA in a protein to the same IAA in a reference pattern of human requirements. To account for differences in bioavailability, the amino acid score (AAS) is corrected for protein digestibility, which must be either obtained from a database or determined by an in vivo assay for true fecal protein digestibility (TFPD) in rats [4]. For example, for soy protein isolate, the AAS is 0.94 with sulfur amino acids being first limiting, the TFPD is 98%, and the PDCAAS is 0.92 [4].

Although the PDCAAS of animal proteins is at or near 1.0, the PDCAAS of plant and other novel proteins is generally lower [5]. Exceptions include soy protein isolate, isolated potato protein, and the mycoprotein from *Fusarium venenatum*, which are also at or near 1.0 [6]. The AAS is the more significant determinant of protein quality because, whereas most proteins are relatively well digested (>75%), most nonanimal proteins contain low ratios of 1 or more IAAs [7]. Compared with animal proteins, the poorer digestibility of most nonanimal proteins can be ascribed to reduced bioavailability because of the presence of cell walls and antinutritional factors, such as antiproteases and tannins [8]; digestibility is improved by thermal or other types of processing that remove these factors [9,10].

Because the PDCAAS of nonanimal proteins is generally lower than that of animal proteins, foods based on nonanimal proteins may be unable to carry a protein content claim; when they do, it is usually a “source” or “good source” of protein claim. Conversely, foods based on animal proteins may support higher content claims (i.e. “high” or “excellent source” of protein), even when the amount of protein in comparable foods based on nonanimal proteins is the same. Furthermore, product-specific data may be unavailable for nonanimal proteins, and in vivo testing to determine digestibility may discourage some consumers from choosing foods when content claims are made based on animal-derived data. This situation raises concern that the requirement to assess protein quality disadvantages foods based on nonanimal proteins, and, in the case of plant-based foods, contrasts with the recommendations of dietary guidelines to increase their consumption [11,12].

On 10 December, 2024, the Physicians Committee for Responsible Medicine, a nonprofit organization dedicated to effective and ethical scientific research and nutrition, hosted a roundtable discussion where participants with expertise in nutrition science, clinical epidemiology, and food regulation weighed the benefits and costs of requiring the in vivo derived PDCAAS to substantiate protein content claims in the United States and Canada. They also considered alternative regulatory approaches to support these claims that could better promote human health and prevent chronic disease, replace animal testing, and support sustainable food production. Options included relying solely on the amount of protein per serving, correcting only for the AAS, using fixed coefficients of digestibility (FCDs) or in vitro assays to determine digestibility under the current framework when digestibility data are unavailable, and incorporating measures that reflect human health outcomes and environmental impact [5,7].

Because determining protein quality will continue to be required for foods that address special needs, such as infant formula or medical/therapeutic foods (e.g. tube feeding formulas), participants concluded that gaining regulatory acceptance of several in vitro assays that are currently undergoing validation should be prioritized. Participants acknowledged that higher protein intakes may benefit specific outcomes in certain subgroups, including greater gains in lean body mass and strength in athletes and preservation of lean mass, swifter recovery from illness, and improvements in immune status, wound healing, blood pressure, and bone health in older adults [[13], [14], [15]]. However, they also concluded that relying solely on the amount of protein to substantiate content claims is appropriate for general populations who already consume protein in excess of daily reference values [e.g. the estimated average requirement, 0.66 g/kg body weight/d, or recommended dietary allowance (RDA), 0.83 g/kg body weight/d [8]] from varied sources [16].

## Currently Required Methods for Determining Protein Quantity and Quality to Support Protein Claims in the United States and Canada

Roundtable participants began the discussion by reviewing current regulatory requirements in the United States and Canada, and the test methods used to determine the total and corrected amounts of protein contained in foods.

To estimate the amount of protein contained in foods, the nitrogen content is multiplied by 6.25, a factor based on the assumption that protein is 16% nitrogen [2,17]. Although the use of specific nitrogen-to-protein conversion factors has been proposed, no consensus method for their determination has been accepted [18]. Nitrogen content is routinely determined by the Kjeldahl or Dumas methods [19]. In the Kjeldahl method, food samples are digested with sulfuric acid, liberating nitrogen as ammonium sulfate, which is converted to ammonium borate. The concentration of hydrogen ions needed to neutralize the ammonium borate solution is equivalent to the nitrogen content [20]. In the Dumas method, food samples are combusted in the presence of oxygen, liberating carbon dioxide, water, and nitrogen, and the carbon dioxide and water are removed by absorption. The nitrogen content is determined by thermal conductivity [21]. Because the nonchemical Dumas method has been automated, it has replaced the Kjeldahl method for determining the nitrogen content of most foods.

In the United States, protein content claims also trigger a requirement to report the percent daily value (% DV) on the Nutrition Facts Label. The DV for protein is 50 g/d, which is based on 10% energy from protein within the context of a 2000 kcal diet [22]. The amount of protein in a food relative to the DV is calculated by multiplying the amount of protein contained in a serving by the PDCAAS and dividing by the DV for protein [2]. Foods that contain 10% to 19%, or >20%, of the DV per reference amount customarily consumed can make a “good source” of protein or “high” protein claim, respectively [23]. Claims that communicate a specific amount of protein are considered expressed content claims and must also meet % DV thresholds [24]. When no content claim is made, the % DV does not need to be reported on the Nutrition Facts label because protein intake is not a public health concern [25]. For the same reason, there is no % DV for protein in Canada [12]. Instead, content claims are based on the protein rating, which is calculated by multiplying the amount of protein in a reasonable daily intake by the PDCAAS [17]. A rating of 8 or 16 qualifies foods for a “source” or “excellent source” of protein claim, respectively [26].

To determine the amino acid composition of a food, 3 hydrolysates are prepared: oxidized protein under acidic conditions (for all amino acids except tryptophan, methionine, and cystine), unoxidized protein under acidic conditions (for methionine and cystine), and unoxidized protein under alkaline conditions (for tryptophan). Amino acids are detected by ion exchange or HPLC. To calculate the AAS, a reference pattern of 9 IAAs plus tyrosine and cystine for children aged 2 to 5 y is used [4]. Participants noted that selection of the reference pattern significantly influences the AAS, with a pattern for older children, adolescents, and adults giving higher scores [27].

To determine the TFPD, groups of ≥4 male weanling rats are fed either a test diet or a protein-free diet for a total of 9 d. The nitrogen content of the food and feces is multiplied by their respective weights, and fecal nitrogen for rats fed the protein-free diet is subtracted from fecal nitrogen for rats fed the test diet. The TFPD is calculated by dividing the corrected fecal nitrogen by the nitrogen intake [4].

## In Vitro Assays for Determining Protein Digestibility

Although the number of animals used per in vivo assay is relatively small, switching to in vitro assays to determine digestibility would significantly reduce animal use. In Canada alone, for which data are available, 2091 high/added protein products were launched between January 2019 and 14 May, 2024 [28]. Conducting in vivo assays to determine the digestibility of these products could have required as many as 16,728 animals. In addition, the use of in vitro assays avoids potential errors arising from microbiome-mediated nitrogen metabolism in the large intestine when determining fecal nitrogen in vivo. Although the digestive enzymes required are currently derived as byproducts from animals used for food production, recombinant human enzymes could potentially better model human digestion. Because in vitro assays are economical and readily implemented, they allow digestibility to be determined for more plant and novel proteins, thereby potentially facilitating and expediting innovation. Several in vitro assays have been reviewed recently, and ongoing collaborative studies evaluating their suitability as alternatives to the in vivo assay have been reported [29].

Mono-compartmental models include the pH-drop and pH-stat methods, both of which are based on the decrease in pH produced by peptide bond cleavage when proteins are digested with trypsin, chymotrypsin, and additional proteases. In the pH-drop method, the change in pH of the digestate over a 10-min incubation period is measured, whereas in the pH-stat method, the volume of base added to maintain its pH at 8.0 is measured. Both methods have a long history of use, and available evidence shows good agreement with the in vivo assay [30]. The Richardson Center for Food Technology and Research served as the Central Laboratory for a ring trial of these methods with 8 additional Canadian and United States laboratories evaluating their accuracy, precision, and interlaboratory reproducibility. Testing of a range of plant and animal proteins for which in vivo assay data were available led to the approval of consensus methods by the Uniform Methods Committee of the American Oil Chemists’ Society in 2024 [31,32].

Multicompartmental models include the Animal-Safe Accurate Protein (ASAP) Quality Score method and the INFOGEST method. In the ASAP-Quality Score method, which is commercially available as the K-PDCAAS kit from Megazyme, a 2-step digestion is used, first by pepsin to simulate the gastric phase and then by trypsin and chymotrypsin to simulate the intestinal phase. α-Amino groups are reacted with ninhydrin, and the absorbance is measured by spectrophotometry [33]. In the INFOGEST method, a third digestion step is used, by salivary amylase to simulate the oral phase [34]. After each step, digestibility can be evaluated by determining total nitrogen, amines, or amino acids, or by measuring the degree of hydrolysis. The INFOGEST method can also be used to determine the true ileal digestibility of amino acids to derive the Digestible Indispensable AAS (DIAAS), for which a ring trial is underway. A validation workflow has been approved, and a joint International Dairy Federation/International Standards Organization protocol is under development [29]. Proposed by the FAO of the United Nations in 2013, the DIAAS accounts for the metabolic effects of individual amino acids [35]. However, it is not yet used in any jurisdiction, and the advantage of transitioning to the DIAAS to substantiate content claims for foods that do not address special needs is questionable [36].

True ileal amino acid digestibility can also be determined in humans and other animals, most commonly in cannulated pigs; however, none of these methods are practical for routine use, and only the INFOGEST method avoids ethical concerns [8]. To overcome these challenges, the FAO of the United Nations, in collaboration with the International Atomic Energy Agency, has initiated the development of a database to include comprehensive data on the protein content, amino acid composition, and ileal digestibility of proteins and individual amino acids in foods, collected by all available methods [37]. Foods based on animal, plant, and other novel proteins will be included, and access will be free of charge [8]. Although the DIAAS was designed to assess the protein quality of single foods [38], true ileal digestibility of amino acids and IAA levels in individual ingredients can be combined to calculate the DIAAS of mixed meals [8]. Nevertheless, a continuing role is envisioned for in vitro methods to screen large numbers of proteins, including different plant varieties, processing and preparation methods [8].

Because the pH-drop and pH-stat methods have been independently validated, they are currently the most promising candidates for regulatory acceptance. Although regulatory change will be required in both the United States and Canada, acceptance may be more readily achieved in Canada, where the requirement for the in vivo assay is contained in an “incorporated by reference” document, for which the revision process is expedited [17].

## Dietary Guidelines on Protein, Saturated Fat, and Fiber

More than half of United States adults have ≥1 preventable chronic health conditions, such as cardiovascular disease (CVD), type 2 diabetes (T2D), obesity, cognitive decline, and colorectal and breast cancer, which are related to unhealthy dietary intakes; diets characterized by higher intakes of plant foods are associated with lower risks for these conditions [39]. Roundtable participants summarized the results of prospective cohort studies of United States health care professionals, which showed that higher intakes of animal proteins were associated with higher CVD mortality, whereas higher intakes of plant proteins were associated with lower CVD and all-cause mortality. In addition, replacing 3% of energy intake from various animal proteins with plant proteins was associated with a 30% reduced risk of all-cause mortality for processed red meats [40]. Higher intakes of animal proteins were also associated with increased risk of T2D, whereas higher intakes of plant proteins were associated with reduced risk. Replacing 5% of energy intake from animal proteins with plant proteins was associated with a 23% reduced risk of T2D [41]. For these reasons, dietary guidelines recommend replacing animal protein sources, especially processed or high-fat animal products, with plant protein sources [11,12].

The association between consumption of saturated fat and risk for CVD has long been understood, and evidence for the deleterious effects of saturated fat on total blood cholesterol and LDL cholesterol is well-established [39]. Compared with animal protein sources, plant protein sources are not only lower in saturated fat but also contain fiber, micronutrients, and potentially beneficial phytochemicals; higher fiber intake is also associated with lower risk of CVD [39].

In both the United States and Canada, saturated fat is a public health concern as 71% of United States females, 76% of United States males, and 50% of Canadian adults consume more than the recommended intake [11,12]. In the United States, fiber is also a public health concern, with 90% of females and 97% of males consuming less than the recommended intake [11]. As noted above, protein is not a public health concern in either jurisdiction, with overall intakes of protein foods close to target amounts in the United States [11]. However, although about three-quarters of United States adults meet or exceed recommended intakes for meats and eggs, more than half do not meet the recommended intakes for nuts, seeds, and soy products; legumes (beans, peas, and lentils), a subgroup of both the vegetable and protein food groups, are also underconsumed [11]. Choosing plant protein sources could help consumers meet recommended intakes for these food subgroups while lowering saturated fat and increasing dietary fiber, potentially lowering CVD and all-cause mortality and decreasing risk of T2D.

## Alignment of Regulatory Requirements with Dietary Guidelines

By communicating nutrition information at the point of purchase, content claims guide consumers in choosing foods [42,43]. Roundtable participants highlighted recent surveys showing that “good source” of protein claims or claims expressing the specific amount of protein are among the most important product descriptions to consumers [[44], [45], [46], [47], [48]]. However, as discussed, requiring the PDCAAS to substantiate such claims disadvantages plant protein sources. For example, a study to determine the PDCAAS of 9 different cooked beans, peas, and lentils found that only navy beans, with a PDCAAS of 0.667, met the 10% DV per serving threshold needed to qualify as a “good source” of protein in the United States [49]. In addition, crop variability and processing can alter the digestibility of plant proteins, necessitating their determination for end products rather than the use of available “historic” data [27,49]. Finally, consumers who avoid products derived from animals for ethical reasons may also avoid foods based on plant and other novel proteins for which protein content claims are made because animals are used to determine digestibility, and organizations advocating for replacing animal testing may not endorse them [7]. Although acknowledging the value of assessing protein quality, roundtable participants expressed concern that requiring the in vivo derived PDCAAS for digestibility perpetuates regulatory barriers to the broader adoption of plant foods.

As discussed, most proteins are relatively well digested, whereas most nonanimal proteins have lower ratios of 1 or more IAAs (mg/g protein) relative to requirements [7]. Therefore, it has been suggested that correcting only for the AAS could be a reasonable approach for qualifying foods for protein content claims [7]. However, roundtable participants noted that although the RDA for protein, which is already intended to meet or exceed the needs of 97% of the population, is 0.83 g/kg body weight/d, United States adults typically consume ∼1.1 to 1.3 g/kg body weight/d [50]. Because this higher intake effectively compensates for the lower quality of some proteins, individuals consuming adequate, varied diets—including vegetarian and vegan diets—will generally meet their required intakes for total protein and all IAAs, and correcting for the AAS may provide little benefit [16]. Subsequent to the roundtable discussion reported in this manuscript, a study was published quantifying protein intake and quality among 193 New Zealand vegans [51]. Although confirming that average IAA intakes were above daily requirements, upon correction for true ileal digestibility (using values from the literature), only ∼50% of the cohort met requirements for lysine and leucine. (Per Table 2 in the study, those in the inadequate protein group were also underconsuming or underreporting calories.) The authors suggest that an increased proportion of legumes and pulses could potentially increase these intakes.

For those with higher protein requirements, such as pregnant and lactating females, and limited access to varied plant-based foods, participants recommended placing greater emphasis on combining plant proteins from different sources to achieve a higher overall AAS. For example, although grain proteins may have low ratios of lysine but relatively high ratios of methionine, legume proteins tend to have the reverse. Many familiar dishes already combine grains with beans, peas, or lentils. In addition, although more research is needed, participants generally agreed that such complementary proteins need not be consumed at the same meal, allowing protein complementarity to arise effortlessly from a variety of plant foods eaten throughout the day [52].

In addition to using in vitro assays to determine digestibility, another option that addresses the needs of individuals with higher protein requirements is using fixed FCDs when digestibility data are unavailable. The TFPD of many plant proteins is >75%, and that of some legumes and nuts is ≥85% [7,53,54], suggesting an appropriate range for FCD values. However, because the digestibility of some isolated plant proteins, such as pea and soy protein, is >98%, roundtable participants observed that producers of plant-based foods using these proteins may prefer to determine digestibility or use available data, rather than underestimate values [7].

Instead of requiring the assessment of protein quality, it is relevant to note that other jurisdictions, including Australia and New Zealand, the European Union, and China and South Korea (which align with Codex Alimentarius guidelines), rely solely on the amount of protein, in either a serving or daily recommendation or per unit of energy, to substantiate content claims [55]. This alternative approach allows more plant-based foods to qualify for protein claims. For example, because they provide >5 g/serving or 20% of energy as protein, all 9 of the beans, peas, and lentils evaluated in the study cited above by Nosworthy et al. [49] would qualify as “source” of and “high” protein foods in Australia and New Zealand and in the European Union, respectively. Other protein foods that currently do not qualify for claims under protein quality assessments implemented by the United States and Canada for protein claims, such as nuts and seeds, would also qualify for claims in these regions. This less burdensome approach aligns with dietary guidelines and avoids animal testing by requiring only the nitrogen content to be determined, further lowering regulatory barriers.

A recent study modeled the effects of protein claim regulatory frameworks from Canada, the United States, Australia, and New Zealand, and the European Union on consumption of plant protein foods using Canadian dietary surveillance data [56]. Protein intake of dietary patterns was high across all diets (>74 g/kg body weight/d) for individuals consuming ≥1 plant protein food that qualified for a protein claim under each framework. Using a conservative digestibility coefficient of 0.8, the protein quality (DIAAS) of diets was also high at >0.94 because individuals consumed animal and plant protein foods. However, plant protein intake increased fiber, calcium, and iron intakes, while lowering saturated fat intake. Importantly, substantially more individuals (*n* ≥ 9578) consumed plant proteins using the Australia and New Zealand and European Union protein claim frameworks compared with the Canadian (*n* = 0–278) and United States groups (*n* = 164). The results suggest that less restrictive regulatory frameworks for protein claims could facilitate better alignment with dietary guidelines and intakes of plant protein foods.

Although studies have yet to show an increase in misleading protein content claims or unmet requirements for intakes of protein or IAAs in these jurisdictions, roundtable participants suggested that studies addressing these concerns are needed. Adopting a similar approach in the United States and Canada would increase intakes of fiber and micronutrients, promoting health and preventing disease [55,56].

As discussed, roundtable participants acknowledged the value of assessing protein quality, especially when protein intake is close to reference values, access to varied plant foods is limited, and no animal protein is consumed, as well as for at-risk populations [16]. To promote health and prevent disease and to support sustainable food production while retaining the requirement to assess protein quality, participants also proposed expanding the definition of protein quality to incorporate measures that reflect human health outcomes and environmental impact. For example, measures that reflect human health outcomes could be based on whether foods are recommended or discouraged in dietary guidelines, and measures that reflect environmental impact could be based on greenhouse gas emissions and water utilization [5]. Together with the AAS and in vitro digestibility or FCDs, incorporating such measures represents a more comprehensive approach to assessing protein quality; however, reaching consensus on the appropriate basis for each, as well as on their ranges of possible values and relative weight, could prove challenging.

In conclusion, by requiring the assessment of protein quality to substantiate protein content claims, current food labeling regulations in the United States and Canada help to ensure that individual foods provide a minimum level of digestible protein relative to IAA requirements. However, basing this assessment on the in vivo derived PDCAAS perpetuates barriers to the broader adoption of plant-based foods. Relying solely on the amount of protein per serving could better align with dietary guidelines, facilitate amino acid complementation, and promote human health and sustainable food production, while avoiding animal use in testing. This approach is appropriate for populations who already consume protein in excess of reference values from varied sources. Although gaining regulatory acceptance of in vitro assays to determine digestibility or the use of FCDs should be prioritized, relying on the amount of protein to substantiate protein content claims, along with incorporating additional measures of quality that reflect human health outcomes and environmental impact, should also be considered.

## Author contributions

The authors’ responsibilities were as follows – JM: contributed to writing—original draft, final content; CDG, AH, ESK, CPFM, AGAS, MS: contributed to writing—review and editing; and all authors: read and approved the final manuscript.

## Declaration of generative AI and AI-assisted technologies in the writing process

During the preparation of this work, JM used ChatGPT (OpenAI) to refine phrasing and NotebookLM (Google) to organize key points. After using these tools/services, JM reviewed and edited the content as needed and takes full responsibility for the content of the publication.

## Funding

The authors reported no funding received for this study.

## Conflict of interest

The authors report no conflicts of interest.
